# Stigmasterol mitigates estrogen-deficiency-induced osteoporosis through inhibition of phosphorylated p65 and MAPK signaling pathways

**DOI:** 10.3389/fphar.2024.1527494

**Published:** 2024-12-16

**Authors:** Qiangqiang Zhao, Xingling Chen, Bin Mai, Feihong Che, Zhen Zhang, Pan Kang, Chengyu Hou, Lu Lu, Liangliang Xu

**Affiliations:** ^1^ Lingnan Medical Research Center, The First Affiliated Hospital of Guangzhou University of Chinese Medicine, Guangzhou, China; ^2^ Lingnan Medical Research Center, Guangzhou University of Chinese Medicine, Guangzhou, China

**Keywords:** stigmasterol, post-menopausal osteoporosis, NF-κB signaling pathway, MAPK signaling pathway, osteoclastic differentiation

## Abstract

**Background:**

Osteoporosis is a pervasive bone metabolic disorder characterized by the progressive degeneration of bone microstructure. Osteoclasts are playing a pivotal role in bone remodeling and resorption. Consequently, modulating osteoclast activity, particularly curbing their overactivation, has become a crucial strategy in clinical treatments. Stigmasterol (STG), a plant-derived phytosterol, has shown promise in inhibiting osteoclastic activity, although its precise biological mechanisms require further scientific investigation. Therefore, this study aims to explore the potential mechanisms by which STG inhibits osteoclasts and to further assess its impact on osteoporosis by establishing an Ovariectomy (OVX) model.

**Methods:**

Initially, osteoclast differentiation was induced *in vitro* using RANKL (Receptor Activator of Nuclear Factor Kappa-B Ligand) on RAW 264.7 cells, followed by TRAP staining and F-actin banding to observe the effects of various concentrations of STG during osteoclast differentiation. The osteoclast-specific gene and protein expression changes were further analyzed using Real-Time PCR (qPCR) and Western blot, exploring the RANKL-mediated NF-κB and MAPK signaling pathways. An OVX model was established *in vivo* to examine changes in bone mass through Micro-CT and Hematoxylin and eosin (H&E) staining, and to assess osteoclast formation and characteristic protein expression through TRAP staining and Immunohistochemistry staining.

**Results:**

*In vitro* experiments revealed that STG significantly inhibited osteoclast activity, as evidenced by reductions in osteoclast numbers and spreading areas, and a marked suppression of F-actin formation. On the molecular level, this compound effectively downregulated key osteoclast markers such as NFATc1, Acp5, c-Fos, and ΜMP9 in both gene and protein expressions. Western blot analysis showed that STG notably inhibited the phosphorylation of the p65 subunit in the NF-κB pathway, thus affecting the pathway’s activity. Further validation through OVX model indicated significant protective effects of STG against bone loss, as demonstrated by Micro-CT. Histopathological staining confirmed STG’s efficacy in reducing bone surface area and volume loss. Additionally, TRAP staining showed significant reductions in osteoclast number and surface area in the STG group compared to the OVX group, underscoring STG’s potential therapeutic role in bone metabolism regulation.

**Conclusion:**

The findings reveal that STG effectively inhibits the phosphorylation of the p65 protein in the NF-κB pathway, and influences the MAPK signaling pathway, thereby reducing osteoclast formation and preserving bone mass. These mechanisms provide a crucial molecular basis for its potential therapeutic application in treating osteoporosis.

## Graphical Abstract

**Figure:**
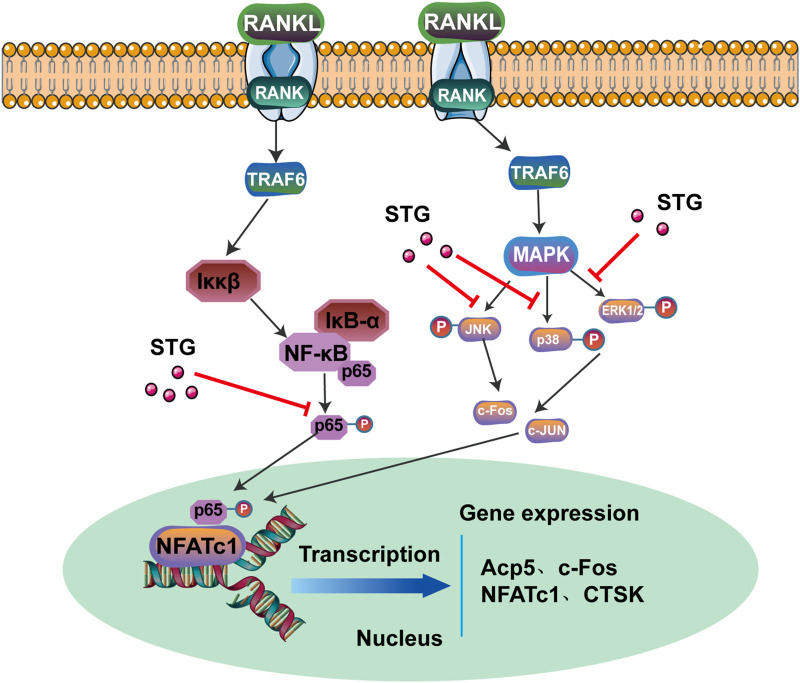

## 1 Introduction

Osteoporosis is a systemic skeletal disorder marked by a reduction in bone mass and the degradation of bone microarchitecture, extensively affecting the global elderly population, particularly postmenopausal women. Pathogenesis of osteoporosis often attributes the reduction in bone mass to the overactivation of osteoclasts and dysfunction of osteoblasts (Da et al., 2021). Osteoclasts dissolve bone tissue through the release of acidic substances and enzymatic activity, while osteoblasts are involved in the formation of new bone during bone modeling and remodeling (Kim et al., 2020). Current therapeutic strategies predominantly focus on inhibiting bone resorption and/or promoting bone formation, utilizing treatments including bisphosphonates and Selective Estrogen Receptor Modulators (SERMs) extensively in clinical settings (Price et al., 2001). However, there remains a pressing need for new therapeutic approaches that enhance bone density and improve bone quality, especially for patients unresponsive to existing treatments.

Stigmasterol (STG), a naturally occurring phytosterol widespread in the plant kingdom, possesses a structural similarity to animal cholesterol. Its molecular formula is C_29_H_48_O, and it contains an unsaturated bond, found in both plant oils and widely distributed in various fruits, seeds, and legumes (Valitova et al., 2024). As an important plant sterol, STG plays a critical role in plant physiological activities and exhibits multiple potential health benefits in human health. In the field of nutrition, STG has garnered attention for its ability to lower blood cholesterol levels. It competes with dietary cholesterol for intestinal absorption, reducing the amount of cholesterol absorbed and consequently lowering blood cholesterol concentration. It has been reported that STG can alter the rheological properties of solid and semi-solid fats, effectively delaying fat oxidation and potentially replacing conventional fats in low-fat food formulations (Xie et al., 2024). Furthermore, STG has been identified to possess anti-inflaμMatory and iμMunomodulatory functions (Jie et al., 2022). At the molecular level, it inhibits the release of key cytokines in inflaμMatory responses, exhibiting anti-inflaμMatory effects (Ma et al., 2024). Previous studies have found that STG effectively inhibits the expression of the acp5 gene mediated by RANKL, playing a role in rheumatoid arthritis, and it also inhibits the expression of inflaμMatory factors such as IL-1β, IL-6, TNF-α, thereby protecting against collagen-induced arthritis (Xie et al., 2023). These findings provide a scientific basis for the application of STG in the development of anti-inflaμMatory drugs. Notably, while the biological activities of STG have prompted extensive research, its specific mechanisms of action and clinical effectiveness still require further verification through more scientific experiments and clinical trials.

In this research, we initially evaluate the impact of STG treatment on RANKL-induced osteoclast formation *in vitro* and ovarianectomy (OVX)-induced bone loss *in vivo*. We also explore the NF-κB and MAPK signaling pathways and elucidate possible molecular mechanisms. Through these investigations, we aim to better understand STG and other natural compounds’ potential in treating osteoporosis and potentially providing more personalized treatment options for patients.

## 2 Materials and methods

### 2.1 Reagents

Stigmasterol (98% purity; Wuhan ChemFaces Biochemical, China) monomers were dissolved in Dichloromethane (DCM, Greagent), making a 10 μM solution that was stored at – 20°C. Thermo Fisher Scientific (Wuhan, China) provided the cell culture media: α-MEM, P/S, and FBS. c-Fos Antibody (c-Fos), CTSK Antibody (CTSK), ERK1/2 Antibody (ERK1/2), JNK1/2/3 Antibody (JNK1/2/3), p38 MAPK Monoclonal Antibody (p38), IKB alpha Antibody (IKBα), Phospho-ERK1/2 (p-ERK), Phospho-JNK1/2/3 (p-JNK), Phospho-p38 MAPK Antibody (p‐P38) and beta Actin Antibody (β‐actin), ΜMP9 Antibody (ΜMP-9) were purchased from affinity biosciences (United States). NFATC1 Antibodies (NFATC1) was purchased from Thermo Fisher Scientific (Wuhan, China). Rhodamine–phalloidin was purchased from Invitrogen (Carlsbad, CA, United States).

### 2.2 Molecular docking

The NFATc1 small molecule protein is downloaded from the PDB database (https://www.rcsb.org). Unnecessary water molecules, ligands, cofactors, and ions are removed using tools such as PyMOL. The 3D structure file of Stigmasterol is obtained from PubChem. (https://pubchem.ncbi.nlm.nih.gov). The ligand file is opened using tools like PyMOL. In AutoDock Tools, configure the ligand as a valid input file. Define the binding site for the ligand in the docking box, ensuring the box encompasses the active site of the macromolecule. The process is run using AutoDock Vina by placing the prepared receptor and ligand files into the specified directory. Finally, the docking results are opened and analyzed using AutoDock Tools (Cai et al., 2021).

### 2.3 Cell culture

Mouse Mononuclear Macrophages Cells (RAW 264.7) were obtained from Thermo Fisher Scientific. The cells were cultured in α-MEM medium supplemented with 10% Fetal Bovine Serum (FBS), in an incubator maintained at 37°C with 5% CO_2_. When cell confluence reached about 90%, the cells were passaged. Cells from passages two to 3 were used for subsequent experiments.

### 2.4 Cell viability assay

RAW 264.7 cells were seeded in a 96-well plate at a density of 10^4 cells per well. STG was diluted into a complete medium to create a concentration gradient of 0, 1, 2.5, 5, and 10 μM, which was then used to treat the RAW 264.7 cells. Forty-8 hours later, the viability of the cells in each well was quantified using an Enzyme-Linked Immunosorbent Assays (ELISA) reader at an absorbance of 450 nm.

### 2.5 *In vitro* osteoclast differentiation assay

Following the research protocol established by Liu et al. (2019), RAW 264.7 cells were seeded at 7 × 10^3 cells per well in a 96-well plate. The cells were treated with 50 ng/mL macrophage colony-stimulating factor (M-CSF) and 50 ng/mL receptor activator of nuclear factor kappa-Β ligand (RANKL) to induce differentiation into osteoclasts. Concurrently, the cells were treated with STG at concentrations of 0, 1, 2.5, 5, and 10 μM. The medium was replaced every 3 days, and the induction was terminated after 6–7 days upon observation of mature osteoclasts. The samples were fixed with paraformaldehyde for a duration of 20 min and subsequently washed three times with PBS. Osteoclasts were stained using a TRAP staining kit (Beyotime, Shanghai, China). Mature multinucleated osteoclasts were imaged using a standard inverted microscope (Olympus, Japan). Finally, osteoclast quantification was carried out utilizing the ImageJ software.

### 2.6 Podosome belt staining

Similar to the TRAP staining procedure, we treated the cells with high and low concentrations of STG (5 and 10 μM) as outlined by Liu et al. (2019). Following fixation and washing of the induced mature osteoclasts, we used the Actin Staining Kit, Alexa Fluor™ Plus 555 (Thermo Fisher Scientific, China), according to the manufacturer’s instructions. Initially, the cells underwent permeabilization with 0.25% Triton™ X-100 in PBS for 10 min; this was followed by incubation with 100 μL of AlexaFluor^®^ 488 phalloidin (1 Unit mixed with 500 μL PBS and 1% BSA, Invitrogen Corp) at room temperature for 20 min. The cells were then washed with PBS and the residual fluid was aspirated. Subsequently, 100 μL of DAPI (Sigma-Aldrich) was added and the cells were incubated for an additional 3–5 min. Imaging was conducted using a fluorescence microscope (Leica, Germany). Multinucleated cells with more than three nuclei were identified as mature osteoclasts. Finally, the quantification of osteoclast numbers and fluorescence intensity was conducted using ImageJ software.

### 2.7 Immunofluorescence for the detection of NFATc1 and p-P65

RAW 264.7 cells were seeded in a 96-well plate and treated with 50 ng/mL macrophage colony-stimulating factor (M-CSF) and 50 ng/mL receptor activator of nuclear factor kappa-B ligand (RANKL), along with STG at concentrations of 5 and 10 μM, similarly to the Podosome belt staining protocol. After permeabilization with 0.25% Triton™ X-100 in PBS. To minimize non-specific antibody binding, the cell surface was blocked using non-specific agents, including BSA or serum. Specific primary antibodies against NFATC1 and phosphorylated P65 were diluted and added to the cells for overnight incubation to allow specific binding to their target antigens. Following this incubation, the cells were washed and incubated with fluorescence-labeled secondary antibodies for 2 h before concluding the experiment. Subsequent washes removed unbound antibodies and impurities. Fluorescent detection involved staining the nuclei with DAPI or similar dyes, facilitating observation under a fluorescence microscope. The cells were then coverslipped to protect them and prevent contamination. Image acquisition was performed similarly to the Podosome belt staining approach.

### 2.8 Quantitative RT-PCR

Following the methodology outlined by Liu et al. (2019), RAW 264.7 cells were seeded in a 6-well plate at a density of 5 × 10^6 cells per well. The cells were treated with 50 ng/mL macrophage colony-stimulating factor (M-CSF) and 50 ng/mL receptor activator of nuclear factor kappa-B ligand (RANKL), and subjected to varying concentrations of STG. The medium was changed every 3 days until mature osteoclasts were observed at days 6–7, at which point the treatment was discontinued. Prior to initiating cell lysis, the growth medium was meticulously removed from the culture dish, and the dish was washed. Then, 1 mL of TRIzol™ Reagent was applied directly onto the cell monolayer in a 3.5 cm diameter culture dish. This volume optimizes the *in situ* lysis of cells. To prevent mRNA degradation, the process proceeded directly to the homogenization of the sample without pre-washing the cells. The cells were thoroughly disrupted using a pipette to ensure a uniform lysate. Following homogenization, the cell lysate was allowed to incubate at room temperature for 5 min to facilitate the complete dissociation of nucleoprotein complexes. For phase separation, chloroform was added at a ratio of 0.2 mL per 1 mL of TRIzol™ used. The tube was securely capped and shaken vigorously to ensure thorough mixing. The mixture was then allowed to settle at room temperature for 2–3 min. Centrifugation was performed at 12,000 × g for 15 min at 4°C, resulting in three distinct phases: a lower phenol-chloroform phase, an interphase, and an upper colorless aqueous phase containing the RNA. The aqueous phase was carefully aspirated while tilting the centrifuge tube at approximately 45° to avoid contamination from the other phases. The RNA-containing solution was then transferred to a sterile tube for further processing. A complete list of primers utilized is provided in Table 1.

**TABLE 1:** Primers for RT-PCR.

Gene	Reverse (5′−3′)	Reverse (5′−3′)	Tm (°C)
c-FOS	GCGAGCAACTGAGAAGAC	TTGAAACCCGAGAACATC	60
CTSK	CTC​GGC​GTT​TAA​TTT​GGG​AGA	TCG​AGA​GGG​AGG​TAT​TCT​GAG​T	60
Acp5	CAC​TCC​CAC​CCT​GAG​ATT​TGT	CAT​CGT​CTG​CAC​GGT​TCT​G	60
NFATc1	GGA​GAG​TCC​GAG​AAT​CGA​GAT	TTG​CAG​CTA​GGA​AGT​ACG​TCT	60
β-actin	GGC​TGT​ATT​CCC​CTC​CAT​CG	CCA​GTT​GGT​AAC​AAT​GCC​ATG​T	60

### 2.9 Western blot assay

Similar to the RT-PCR process, upon observing mature osteoclasts, protein extraction was conducted on RAW 264.7 cells using an appropriate lysis buffer, such as RIPA buffer, supplemented with protease and phosphatase inhibitors to protect the proteins from degradation. The protein concentration was determined through the BCA assay to ensure it was adequate for subsequent SDS-PAGE electrophoresis. Subsequently, the protein samples underwent SDS-PAGE separation, enabling the differentiation of proteins according to their molecular weight. Post SDS-PAGE, the separated proteins were transferred to a solid support, such as a PVDF membrane or a nitrocellulose membrane (NC membrane). The PVDF membrane was washed with TBST buffer, followed by a blocking step to eliminate non-specific binding. Specific antibodies, both primary and secondary, were used to incubate the membrane, facilitating the detection of the target proteins. The membrane was then exposed to an ECL detection reagent to visualize the signal bands corresponding to the target proteins. The intensity of these bands was analyzed to quantify the expression levels of the target proteins.

### 2.10 Ovariectomy was performed to establish an osteoporosis model

This study utilized 32 specific pathogen-free (SPF) C57BL/6J female mice, sourced from the Guangdong Medical Laboratory Animal Center (production license number: SCXK, 2022–0002). The mice, aged 7 weeks and weighing 22 ± 3 g, were housed under SPF conditions at the First Affiliated Hospital of Guangzhou University of Chinese Medicine (license number: SCXK 2023–0092). The mice were maintained under standard conditions with *ad libitum* access to food and water. The animal experiments were approved by the Animal Experimentation Ethics Committee of Guangzhou University of Chinese Medicine (approval number: 20230417003).

As described previously (Chen et al., 2019), an osteoporosis model was simulated using ovariectomy. Initially, the mice were anesthetized with an intraperitoneal injection of pentobarbital sodium. The mice were then placed in a supine position on a surgical board, and their dorsal fur was removed. A skin incision approximately 0.5–1 cm long was made along the dorsal midline using surgical scissors, and the skin and muscle were bluntly separated. The muscle layer was visible through the incision, and a 1 cm incision was made laterally from the spine below the rib to expose the adipose tissue enveloping the ovaries and the closely attached uterine horns. The adipose tissue was gently grasped and extracted through the incision to locate the cauliflower-like structure of the ovaries. Ligations were performed at the upper and lower portions of the fallopian tubes at the uterine horns. The adipose tissue surrounding the ovaries was peeled off, and the uterine horns were shortened with surgical scissors to remove the ovaries. The adipose tissue was then repositioned into the abdominal cavity, and both the peritoneum and muscle layer were sutured together. The skin was also sutured. Post-surgery, the mice were placed on a thermostatic heating pad to provide a suitable recovery environment. Penicillin was administered during the first week post-operatively to prevent infections, and the wound site was disinfected. The mice were observed for any unusual behaviors or conditions and were then divided into four groups: Control, OVX, OVX + STG (5 mg/kg), and OVX + STG (10 mg/kg). STG was dissolved in corn oil and administered via gavage every other day; the Control and OVX groups received corn oil alone. This process was continued for 8 weeks, during which no adverse reactions or deaths were observed in the mice.

### 2.11 Micro-CT and histomorphometry analysis

Following the conclusion of the medication protocol, an overdose of pentobarbital sodium was administered to the mice to perform euthanasia, and bilateral facial muscle tissues were carefully dissected. Subsequently, these tissues were immersed in a 4% solution of paraformaldehyde (PFA) for 48 h to fix the tissue structures. Thereafter, precise scans of the distal femur were conducted using a high-resolution Bruker Skyscan 1,172 micro-computed tomography (micro-CT) system. The scanning parameters were set as follows: a voltage of 85 kV, a current of 120 μA, a 0.5 mm aluminum filter, and a pixel size of 9 microns. To ensure uniformity of the analysis sites across all groups, we manually selected a region composed of 100 consecutive sections located 0.1 mm below the growth plate, analyzed at the level of the tibia. Bone mineral density (BMD), the ratio of bone volume to total volume (BV/TV), the number of trabeculae per cross-section (Tb. N), and trabecular thickness (Tb. Th) were quantified using CT Analyser software.

Finally, the samples were treated with an EDTA solution for 21 days to decalcify, followed by paraffin embedding. Slices were prepared using a Leica HistoCore RM2265 rotary microtome, set at a thickness of 6 μm. Once the sections were cut, the machine was zeroed. The sections were then baked for approximately 3 days at 36°C or heated for 2 h at 60°C in a slide warmer, and stored in a dry environment for subsequent experimental assessments.

### 2.12 Statistical analysis

In this study, all assays were performed in triplicate at a minimum. Statistical analysis was conducted using GraphPad Prism 9.0 software (San Diego, United States). The results are displayed in bar graphs, representing mean values ±standard deviation (SD). The statistical significance of differences among groups was assessed using one-way ANOVA, with a threshold P-value of <0.05 indicating statistical significance.

## 3 Results

### 3.1 STG inhibited osteoclast differentiation in a concentration-gradient manner

**FIGURE 1:**
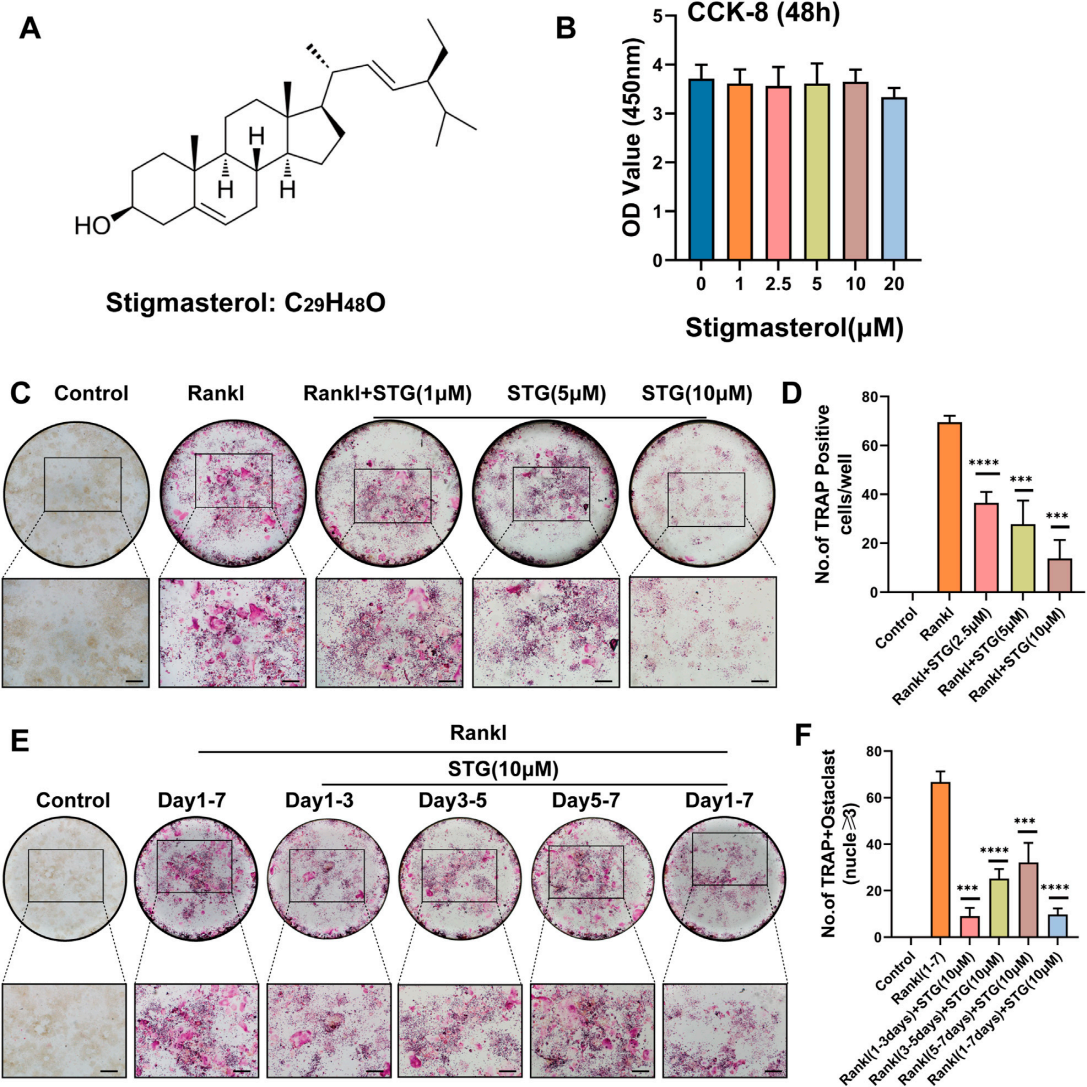
STG inhibited RANKL-induced osteoclast differentiation. **(A)** Molecular and structural formula of STG. **(B)** The CCK-8 assay was used to investigate the effects of various concentrations of STG on the viability of Raw cells. **(C, D)** TRAP staining illustrated the effects of STG at different concentrations (0, 1, 2.5, 5, and 10 µM) on osteoclast differentiation, accompanied by quantitative analysis. **(E, F)** TRAP staining and quantitative analysis were performed to examine the impact of STG on osteoclast maturation across several time intervals. Scale bar = 200 µm. Data are presented as mean ± SD. Each group consisted of three samples (n = 3 per group). “ns” indicates no statistical significance. Significance levels are denoted as *p < 0.05, **p < 0.01, ***p < 0.001, ****p < 0.0001.

Figure 1A displays the molecular structure of STG. To investigate whether STG exerts cytotoxic effects on the proliferation of RAW 264.7 cells, we treated the cells with various concentrations of STG for 48 h, as depicted in Figure 1B. Results from the CCK-8 assay indicated that cytotoxicity did not significantly change within concentrations ranging from 0 to 10 μM; however, cellular viability decreased at 20 μM. Therefore, we selected the highest non-cytotoxic concentration of 10 μM for further cellular experiments. Based on these results, we examined whether STG could inhibit the maturation and differentiation of osteoclasts using TRAP staining. Initially, RAW 264.7 cells were treated with ST G at concentrations of 2.5 μM, 5 μM, and 10 μM, along with 50 ng/mL M-CSF and 50 ng/mL RANKL, over a period of 6–7 days. Treatment was stopped upon observation of mature osteoclasts. Cells not treated with STG exhibited a higher number of mature osteoclasts. After treatment with STG, there was a dose-dependent inhibition of osteoclast formation, with the most significant inhibition observed at 10 μM (Figure 1C). Statistical analysis corroborated these findings, as shown in Figure 1D. Furthermore, to more directly observe the effects of STG on osteoclast differentiation over different periods, we conducted inductions and stainings at varying timelines. We noticed that STG exhibited a more pronounced inhibitory effect on the number of positive osteoclasts in the early phase (1–3 days) compared to later stages (Figures 1E, F), indicating that STG primarily interferes with the early stages of osteoclast differentiation. In summary, these findings reveal that STG inhibits the maturation of osteoclasts in a concentration-dependent manner, with primary effects occurring during the early stages of osteoclast maturation. Recently, STG has been identified as having potential inhibitory effects on osteoclasts from various aspects.

### 3.2 STG inhibited the formation of F-actin in osteoclasts

**FIGURE 2:**
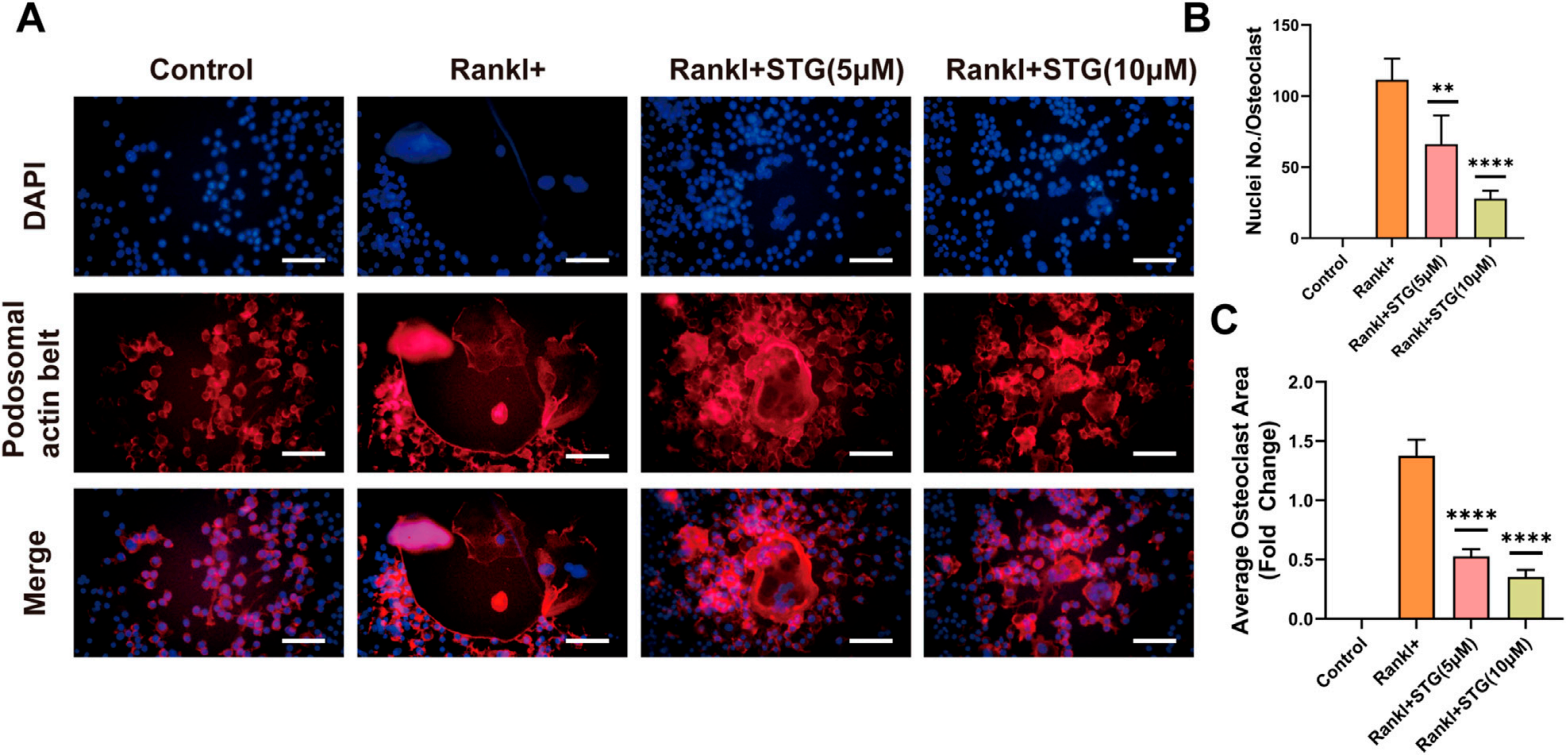
STG inhibited the formation of the characteristic marker “Podosome belt staining” in osteoclasts. **(A)** Formation status of osteoclasts following treatment in the Control, Rankl, STG (5 µM), and STG (10 µM) groups. **(B–C)** Quantitative analysis of the number of nuclei and surface area associated with the formation of Podosome belt staining in Raw cells. Scale bar = 100 µm. Data are presented as mean ± SD. There were three samples in each group (n = 3 per group). Significance levels are indicated as follows: *p < 0.05, **p < 0.01, ***p < 0.001, ****p < 0.0001.

As F-actin rings are characteristic markers of osteoclast maturation, we aimed to examine the effects of STG on their formation. To this end, we selected 5 μM and 10 μM as low and high concentrations of STG for testing. Following the induction treatment, we conducted staining with rhodamine-labeled phalloidin. Similar to the results observed with TRAP staining, STG exhibited a significant inhibitory effect on the formation of F-actin, which was dose-dependent (Figure 2A). Further, we performed statistical analysis on the number of F-actin rings and the area covered by F-actin in osteoclasts (Figures 2B, C).

### 3.3 STG downregulated the expression of key genes and proteins in osteoclasts

**FIGURE 3:**
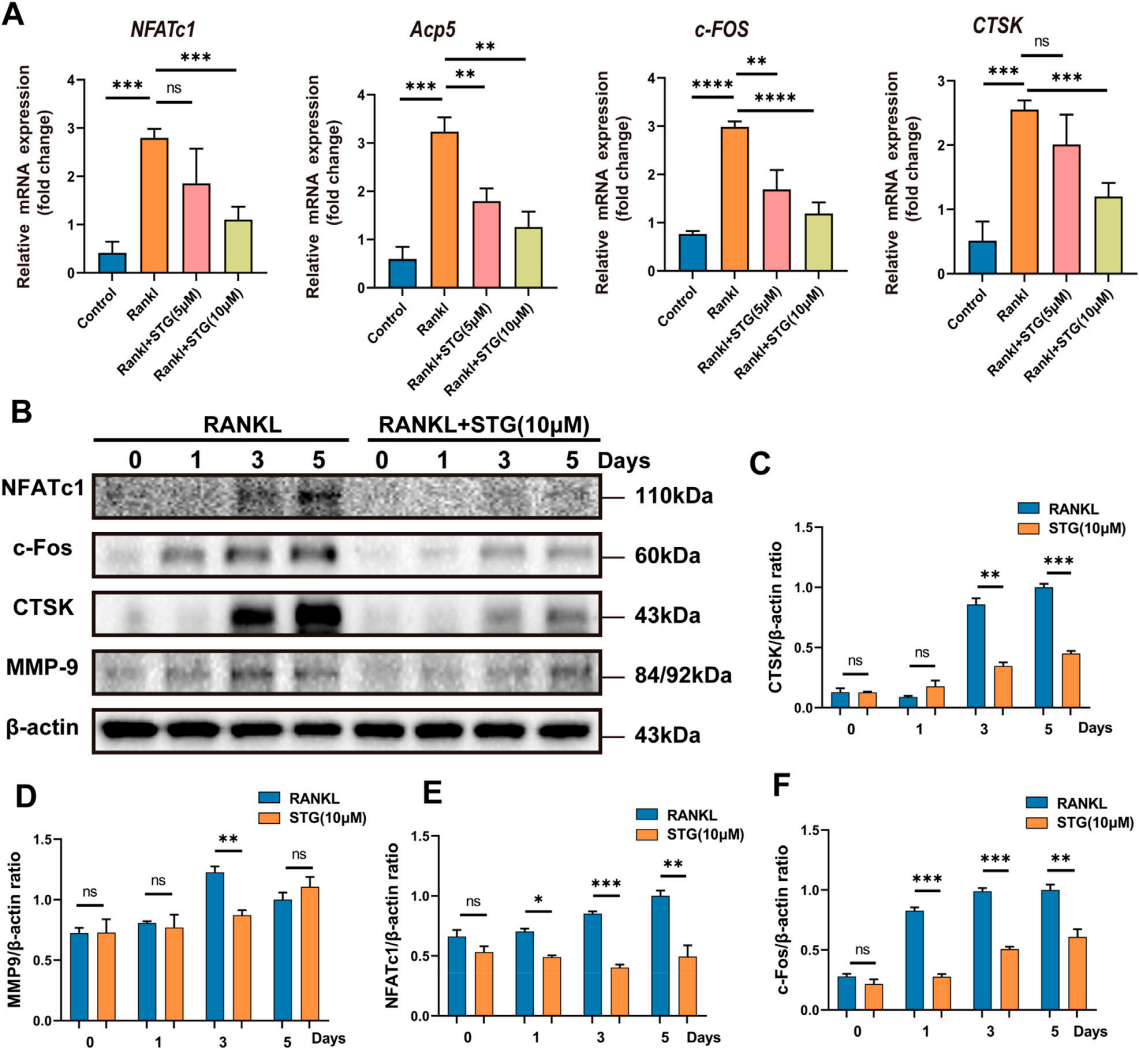
Inhibitory effects of STG on osteoclast-characteristic genes and proteins. **(A)** qPCR analysis was performed to assess the expression of the genes NFATc1, Acp5, c-Fos, and CTSK in osteoclasts across different experimental groups. **(B)** Western blot analysis was conducted to investigate protein expression in osteoclasts induced by Rankl for 6–7 days following STG treatment, along with statistical analysis. Data are presented as mean ± SD. There were three samples per group (n = 3 per group). Significance levels are indicated as follows: *p < 0.05, **p < 0.01, ***p < 0.001, ****p < 0.0001.

Similarly, genes such as NFATc1 and c-Fos play important roles in the differentiation and maturation of osteoclasts. The binding of RANKL to TNF receptor-associated factor 6 (TRAF6) activates transcription factors such as NF-κB and the mitogen-activated protein kinase (MAPK) pathways (Han and Kim, 2019). Once activated, NFATc1 translocates into the nucleus, enabling osteoclasts to function effectively. Consequently, we further explored this process at the genetic level using Real-Time PCR to assess RANKL-mediated osteoclast differentiation and the impact of STG thereon. qPCR results indicated significant upregulation of Acp5, NFATc1, c-Fos, and CTSK genes in RAW 264.7 cells under the influence of RANKL, but this trend was significantly downregulated following treatment with STG at concentrations of 5 and 10 μM, inhibiting the upregulation of these genes (Figure 3A).

Similarly, protein levels were assessed, and, as anticipated, the expression of proteins such as NFATc1 and c-Fos increased akin to gene expression levels under the influence of RANKL. However, these levels significantly decreased after STG treatment, with a dose-dependent enhancement of the inhibitory effect, as evidenced by the statistical analysis (Figures 3B–F). In summary, from various perspectives and at different levels, we validated the pharmacological effects of STG, demonstrating its ability to inhibit osteoclast maturation and differentiation in a concentration-dependent manner. However, the underlying molecular mechanisms and specific targets of the drug remain unclear, prompting further investigation into these mechanisms.

### 3.4 Molecular docking results of STG and NFATc1 and immunofluorescence to detect the expression of NFATc1 protein

**FIGURE 4:**
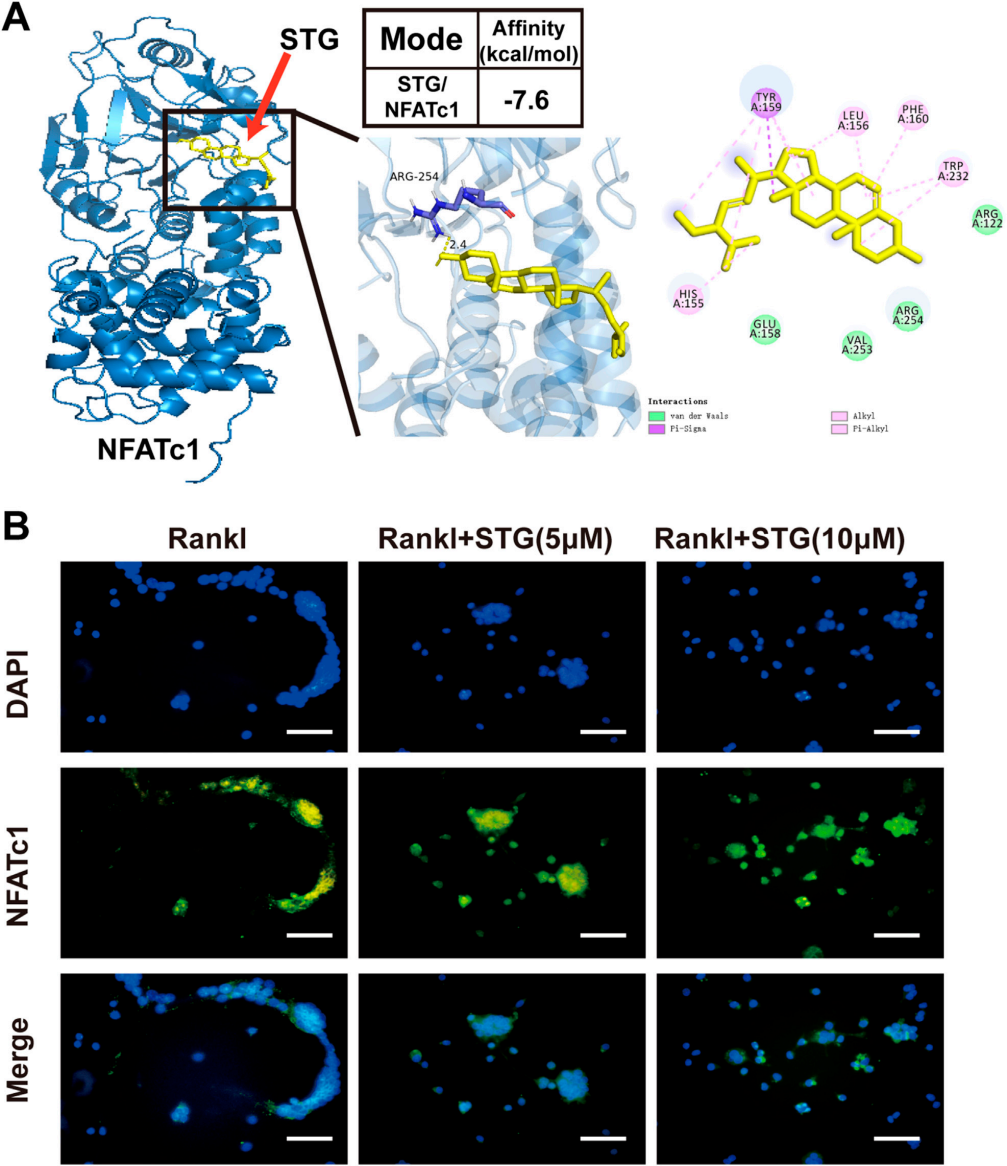
Inhibitory effect of STG on the key protein NFATc1. **(A)** 3D image of the binding of the STG molecule and the NFATc1 protein molecule. The affinity between STG and NFATc1, and the 2D docking plot showing the specific hydrogen bonding sites of STG and NFATc1, respectively. **(B)** The expression of NFATC1, a key protein in osteoclasts, was detected by cell fluorescence after different concentrations of STG. Scale bar 100 µm. The data is represented as mean ± SD. n = 3 per group. *P < 0.05, **P < 0.01, ***P < 0.001, ****P < 0.0001.

As the primary regulator of osteoclastogenesis, NFATc1 controls the expression of osteoclast-specific genes such as tartrate-resistant acid phosphatase, calcitonin receptor, and cathepsin K. Furthermore, STG exhibited inhibitory effects at both the genetic and protein levels. Molecular docking provides a simulation of how small molecules interact with the surfaces of biomacromolecules at an atomic level. This technique is employed to predict possible binding modes and affinities between them, which, in turn, aids in assessing the activity and selectivity of drugs or in elucidating the interactions between drugs and proteins. Therefore, we employed molecular docking techniques to analyze the interaction between the small molecule drug Stigmasterol (molecular formula: C_29_H_48_O) and the protein NFATc1. The results, as shown in Figure 4A indicates a strong binding affinity between STG and NFATc1, with an affinity of −7.6 kcal/mol, suggesting a higher stability of the receptor-ligand complex. Concurrently, we used immunofluorescence technology to examine NFATc1 protein expression in RAW 264.7 cells (Figure 4B). The results demonstrate that under the influence of RANKL, RAW 264.7 cells exhibit significant aggregation and cell wall fusion, forming large osteoclasts. The presence of STG altered this phenomenon, reducing the size and area of osteoclasts and inhibiting their fusion at low concentrations, with more pronounced effects at higher concentrations. Thus, we propose that the mechanism through which STG exerts inhibitory effects on osteoclast activity may entail the suppression of NFATc1 pathway activity, thereby affecting downstream signal transduction and transcriptional activation.

### 3.5 STG inhibited osteoclast differentiation by downregulating key proteins of the NF-κB pathway and MAPK pathway

**FIGURE 5:**
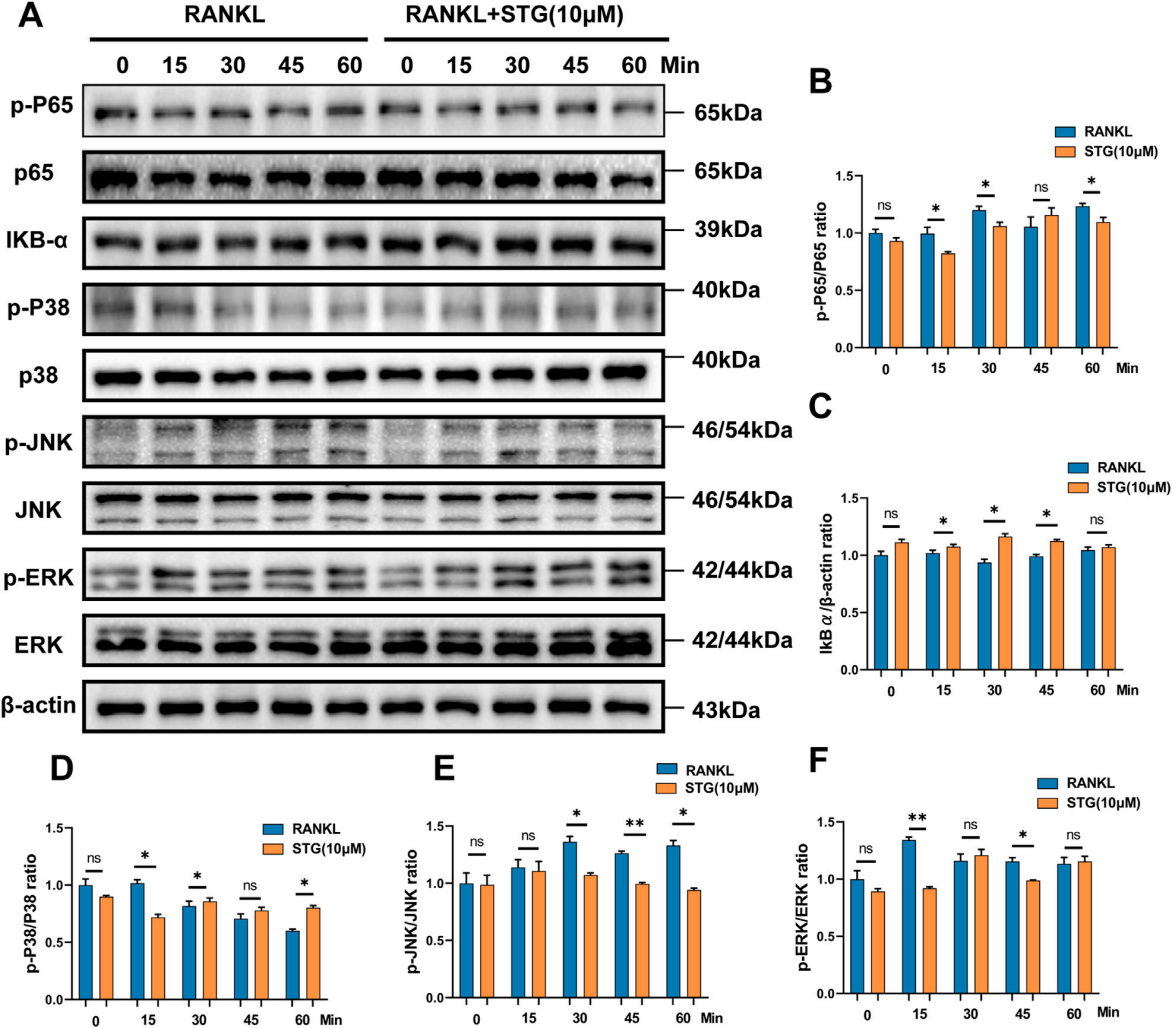
STG inhibited osteoclasts by regulating the NF-κB signaling pathway and MAPK signaling pathway. **(A)** During osteoclastogenesis, STG inhibits the phosphorylation of ERK and JNK within the MAPK pathway, as well as the nuclear transcription of phosphorylated p65 in NF-κB. Treatment with 10 μM STG for 1 h resulted in a decrease in protein expression and phosphorylation of ERK, P38, JNK, iκBα, and P65, as detected by matching antibodies. **(B–F)** Statistical analysis compared the levels of phosphorylation against changes in ERK, P38, JNK, iκBα, and P65. Data are presented as mean ± SD. The sample size was n = 3 per group. Statistical significance is annotated as *p < 0.05, **p < 0.01, ***p < 0.001, ****p < 0.0001.

**FIGURE 6:**
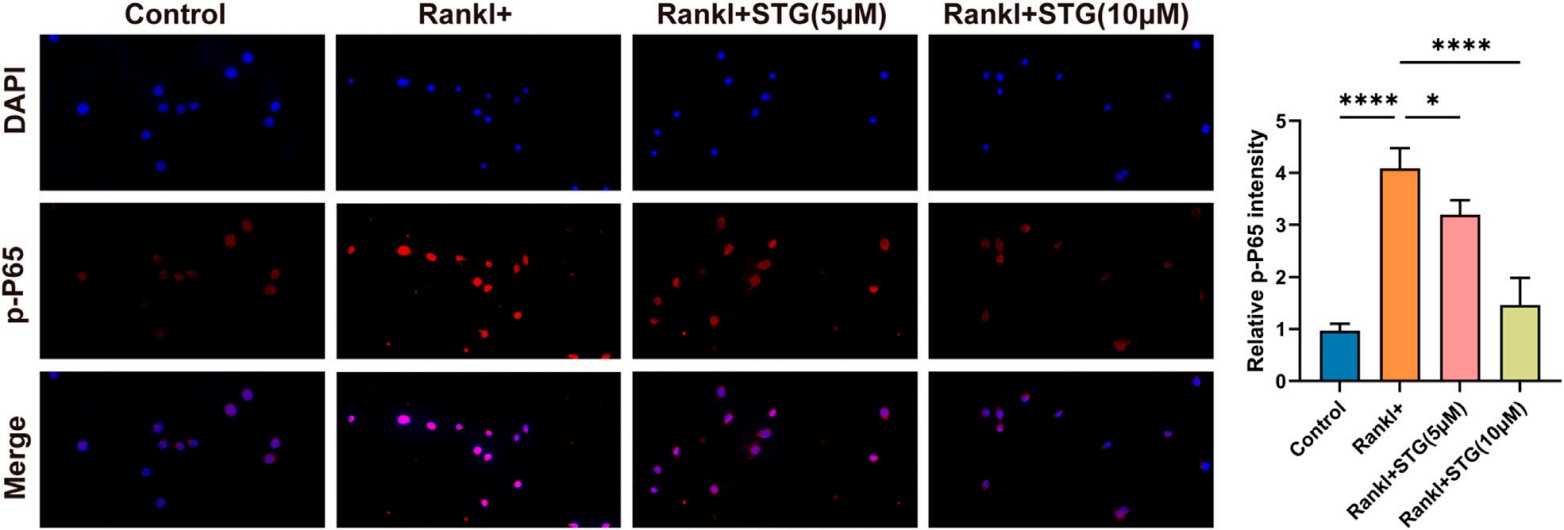
Cellular fluorescence intensity illustrated the change in phosphorylated p65 after STG treatment. Cellular fluorescence intensity demonstrated the expression of phosphorylated p65 in four groups: Control, Rankl, STG (5 µM), and STG (10 µM), with quantitative analysis of fluorescence intensity performed. Scale bar = 100 µm. Data are presented as mean ± SD. There were three samples in each group (n = 3 per group). Significance levels are indicated as follows: *p < 0.05, **p < 0.01, ***p < 0.001, ****p < 0.0001.

Research has demonstrated that the binding of RANK to RANKL activates TRAF6, which in turn activates ASK1 kinase, leading to the phosphorylation of JNK, ERK, and p38, thus activating the MAPK signaling pathway. To explore these findings further, we conducted a study. In the results of our Western blot assay, we observed a surprising trend. Compared to the RANKL group, treatment with STG (10 μM) resulted in a notable decrease in the phosphorylation levels at the 15, 30, and 60-min time points. Conversely, IKKα showed an upregulation at the 15, 30, and 45-min time points (Figure 5A). These observations suggest that STG may inhibit the nuclear translocation of phosphorylated p65, thereby reducing transcription of key osteoclast genes such as NFATc1 and c-fos, potentially uncovering the targets of STG’s activity. We also examined the key proteins in the MAPK pathway. Compared to the RANKL model group, phosphorylation of ERK exhibited a decreasing trend at the 15 and 45-min marks. Phosphorylation of JNK also showed a significant decrease at the 30, 45, and 60-min intervals. However, phosphorylated p38 did not exhibit noticeable effects (Figures 5B–F). In addition, we investigated the fluorescence intensity of phosphorylated P65 following treatment with STG using cellular fluorescence imaging techniques. Compared to the Rankl-treated group, the fluorescence intensity of phosphorylated P65 was reduced after STG treatment, particularly at a concentration of 10 µM (Figure 6). Based on these results, we discovered that STG downregulates phosphorylated ERK and JNK to decrease the transcription of OC genes, thus inhibiting the maturation of osteoclasts. Overall, our study made the remarkable discovery within the RANKL-mediated NF-κB and MAPK pathways that STG effectively inhibits the nuclear translocation and transcription of phosphorylated p65, as well as the expression of phosphorylated ERK and JNK, particularly highlighting the pronounced effect on JNK. Building upon these findings, we discussed the potential role of STG in osteoclast differentiation and uncovered new insights.

### 3.6 STG protects against OVX-induced bone loss

**FIGURE 7:**
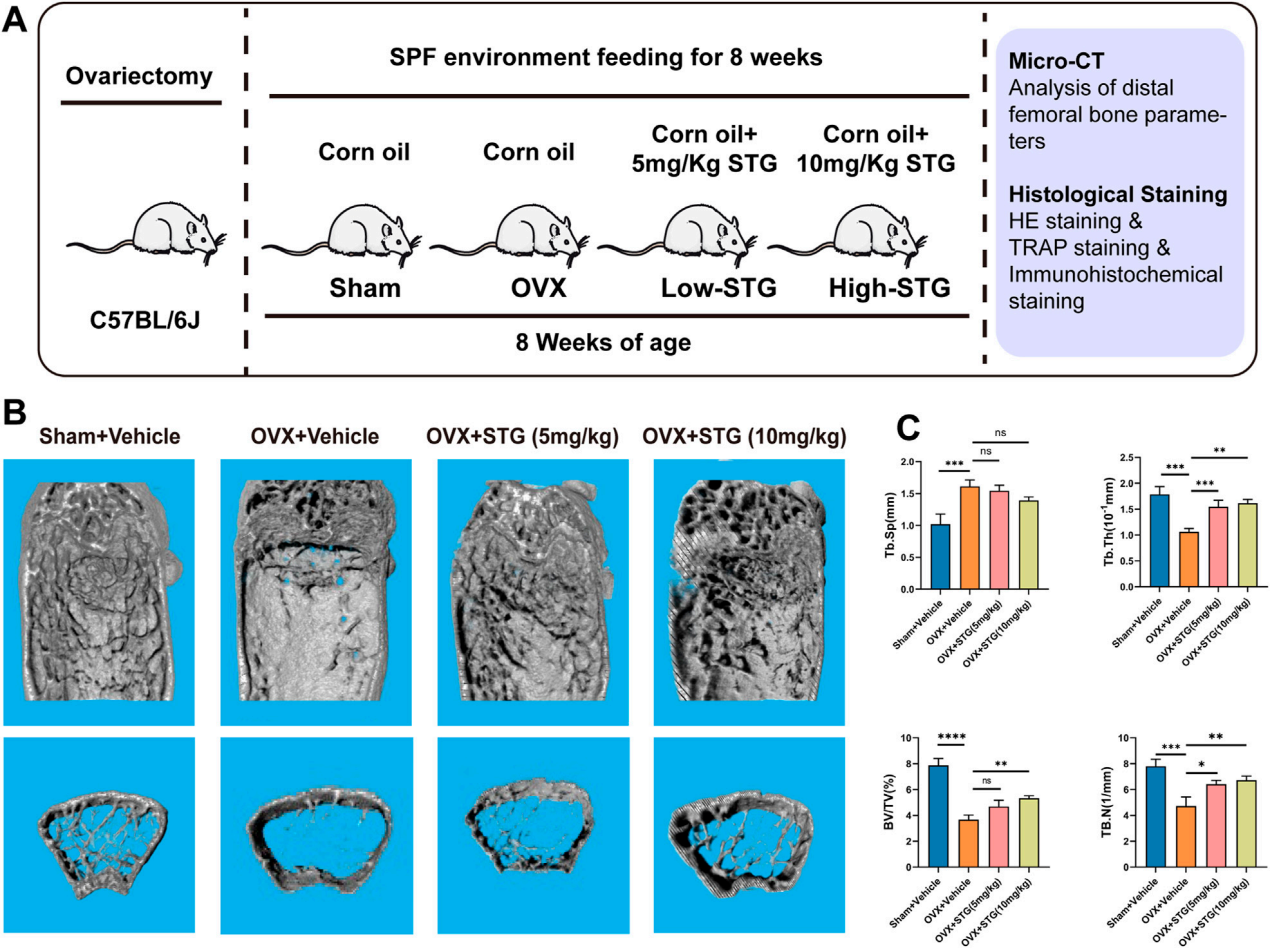
STG mitigated bone mass loss in the OVX model. **(A)** STG application during our *in vivo* experiments. **(B)** Three-dimensional CT images of the distal femur. **(C)** Analysis of bone parameters below the growth plate of the distal femur, including bone volume fraction (BV/TV), trabecular number (Tb. N), trabecular thickness (Tb. Th), and trabecular separation (Tb. Sp) (n = 6). Data are presented as mean ± SD. n = 8 per group. *p < 0.05, **p < 0.01, ***p < 0.001, ****p < 0.0001 relative to the OVX group.

To investigate the potential therapeutic effects of STG *in vivo*, we established an osteoporosis ovariectomized (OVX) model through surgical procedures. We initially determined the optimal oral dosages of STG to be 5 mg/kg and 10 mg/kg. These were administered via gavage dissolved in corn oil, matched with equivalent volumes of corn oil for both the sham and OVX model groups, over 8 weeks (Figure 7A). No mortality or other abnormalities were observed in the mice during the modeling process or the STG treatment period. Micro-CT scanning demonstrated that, compared to the sham group, the OVX procedure led to significant bone loss at the distal femur. However, treatment with STG effectively counteracted this bone loss, with the higher concentration of STG showing more pronounced effects (Figure 7B). Subsequent micro-CT analysis of the distal femur revealed significant improvements in bone metrics, such as bone volume fraction (BV/TV), trabecular number (Tb. N), trabecular thickness (Tb. Th), and trabecular separation (Tb. Sp). The improvements were notable in both the high and low concentrations of STG, with the 10 mg/kg dosage showing the best results (Figure 7C). Further, histological staining allowed for a more direct observation of bone mass changes across the groups.

**FIGURE 8:**
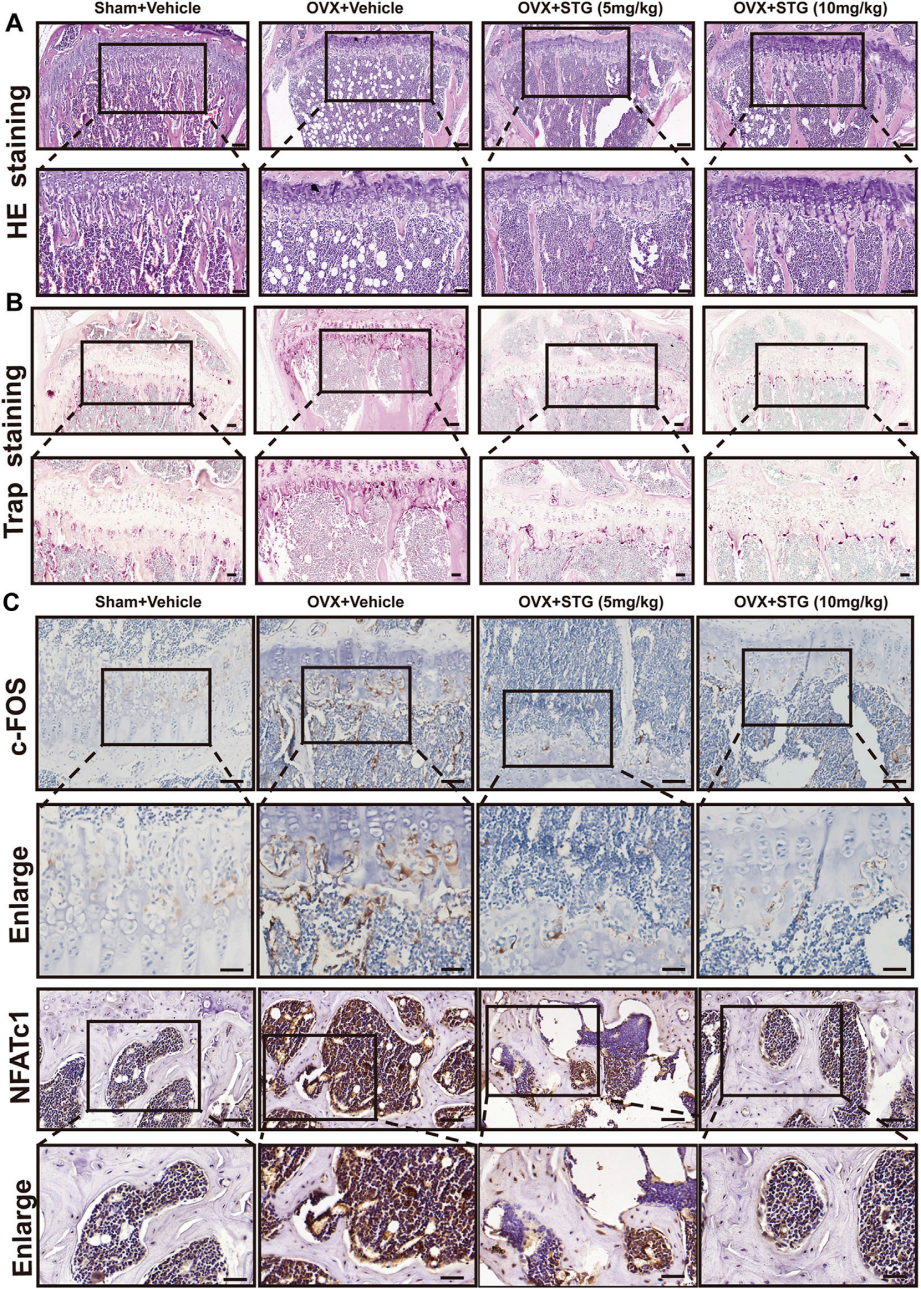
STG reduced bone mass loss in the OVX model by inhibiting osteoclast production. **(A)** HE staining illustrates the changes in bone mass across the groups. **(B)** TRAP staining reveals variations in osteoclasts among the four groups, including their number and area. **(C)** Immunohistochemistry shows the alterations in key osteoclast proteins NFATc1 and c-Fos in the distal femur. Scale bar = 100 or 200 µm. Data are presented as mean ± SD. n = 8 per group. *p < 0.05, **p < 0.01, ***p < 0.001, ****p < 0.0001.

HE staining showed a marked reduction in bone volume beneath the growth plates in the OVX model group, which was noticeably improved after STG treatment, especially in the magnified areas (Figure 8A). TRAP staining indicated that the OVX model group exhibited a higher number and larger area of osteoclasts, while the STG-treated groups demonstrated a significant downward trend in both the number and size of osteoclasts (Figure 8B). Additionally, immunohistochemical staining was employed to assess the expression of key osteoclast proteins NFATc1 and c-FOS in the groups. The results indicated a significant reduction in NFATc1-positive and c-FOS-positive protein expressions in the STG groups, which played a critical role in mitigating bone loss (Figure 8C). In summary, these results demonstrate STG’s inhibitory effects on key proteins in osteoclasts, providing protective effects against bone loss induced by estrogen deficiency following ovariectomy.

## 4 Discussion

In the human skeletal system, bone homeostasis is crucial in influencing bone health and is tightly regulated by two types of cells: osteoblasts and osteoclasts. Under normal physiological conditions, osteoblasts secrete RANKL protein, which acts on osteoclasts by activating the RANK protein on their surface, thereby promoting osteoclast maturation and initiating their bone resorptive function. Additionally, osteoblasts produce osteoprotegerin (OPG), a protein that competes with RANKL for binding to osteoclasts, preventing the imbalance caused by excessive activation of osteoclasts (Boyce and Xing, 2007; Udagawa et al., 2021). Concurrently, the maturation and differentiation of osteoclasts are also influenced by Macrophage Colony-Stimulating Factor (M-CSF) (Boyce, 2013). Under the influence of these ligand proteins and growth factors, osteoclasts develop into mature cells, characterized by the formation of F-actin rings, which are key to their bone resorption function.

In this study, the TRAP staining and Podosome belt staining revealed that STG exhibited a concentration-dependent inhibitory effect on osteoclastogenesis. Additionally, STG downregulated the expression of key genes and proteins involved in osteoclast maturation, including NFATc1, Acp5, MMP9, and c-Fos. Particularly, after STG treatment, the expression of the NFATc1 protein in osteoclasts was markedly suppressed—a finding that was further supported by our molecular docking and cellular fluorescence assays, suggesting that STG may play a crucial role in inhibiting osteoclast formation. Based on these findings, we further explored the molecular mechanisms of STG in the maturation and differentiation of osteoclasts.

NFATc1 is a crucial transcription factor that plays a key role in various processes of bone regeneration, including osteoblast differentiation and osteoclastogenesis (Yu et al., 2022). Particularly in the differentiation of osteoclasts, NFATc1 serves as the primary regulator and executor induced by RANKL (receptor activator for nuclear factor κB ligand), a significant cytokine that promotes the formation and activation of osteoclasts. During this process, NFATc1 upregulates the expression of a set of genes responsible for osteoclast adhesion, migration, acidification, and the degradation of both organic and inorganic bone matrices, thus playing an essential role in osteoclast fusion and activation. For example, NFATc1 was shown to upregulate the expression of the solute carrier family 7 member 11 (SLC7A11) through transcriptional regulation during RANKL-induced osteoclastogenesis (Zhong et al., 2023). Furthermore, serine can drive osteoclast differentiation by epigenetically regulating the expression of NFATc1. Therefore, by modulating the expression of NFATc1, a complex synergistic control mechanism can be established, which aids in maintaining bone homeostasis (Stegen et al., 2024). In this research, we discovered through *in vitro* and *in vivo* experiments that after STG treatment. Moreover, it is noteworthy that the role of NFATc1 is not limited to osteoclast differentiation. Recent studies have also identified NFATc1 expression in articular chondroprogenitor cells, where it acts as a key transcription factor regulating the differentiation of articular cartilage cells. This finding has significant implications for understanding the pathogenesis of joint diseases such as osteoarthritis and for developing new therapeutic approaches.

The central role of the NF-κB signaling pathway in the generation and activation of osteoclasts has been widely recognized (Abu-Amer, 2013; Novack, 2011). Nuclear factor κB (NF-κB), is extensively involved in various cellular activities, including immune responses, inflammation, and cell survival. The NF-κB signaling pathway plays a critical regulatory role in the generation and function of osteoclasts. Originating from the differentiation of bone marrow macrophages, osteoclasts are multinucleated cells tasked primarily with maintaining skeletal remodeling and balance through bone resorption processes (Yu et al., 2021). This intricate process is finely orchestrated by multiple signaling pathways, with NF-κB playing a pivotal role. Upon stimulation by external triggers such as receptor activators for nuclear factor κB ligand (RANKL) and tumor necrosis factor-alpha (TNF-α), the NF-κB pathway is promptly activated (Lawrence, 2009; Fischer and Haffner-Luntzer, 2022). These extracellular signals initiate a cascade of downstream signaling responses by binding to specific receptors, leading to the phosphorylation and degradation of IκB proteins, thereby allowing NF-κB to be released and enter the nucleus to regulate the expression of specific genes. Within the RANKL-induced signaling pathway, the interaction between RANK (the receptor for RANKL) and TNF receptor-associated factors (TRAFs), particularly TRAF6, activates the IκB kinase (IKK) complex, further causing the phosphorylation and ubiquitin-mediated degradation of IκBα (Wang et al., 2018; Jang et al., 2019). IκBα typically binds NF-κB and retains it in the cytoplasm. However, following the degradation of IκBα, the NF-κB dimer (primarily p65/p50) is transported to the nucleus, initiating the expression of genes associated with osteoclastogenesis and function, such as c-Fos and NFATc1 (Yan et al., 2001). The activation of the NF-κB pathway subsequently promotes the activation of c-Fos and NFATc1, which are critical for the early development of osteoclasts (Yamashita et al., 2007). As a primary regulatory factor in osteoclast formation, NFATc1’s expression further enhances the activation of osteoclast-related genes (Zhao et al., 2010). Additionally, the NF-κB pathway also plays a crucial role in conditions such as osteoporosis and rheumatoid arthritis, often associated with excessive or sustained activation of the NF-κB pathway, leading to increased osteoclast activity and bone resorption. Reports suggest that double knockouts of NF-κB genes result in severe osteopetrosis (Yang et al., 2021).

Thorough studies of this pathway not only help reveal the biological mechanisms of osteoclasts but also provide potential targets and approaches for the treatment of bone diseases. In our experiments, we were surprised to find that STG exerts significant inhibitory effects on key proteins in the MAPK and NF-κB pathways at various time points. To establish an osteoclast model, we induced differentiation using RANKL and observed through Western blot experiments that STG significantly inhibits the phosphorylation of p65 at 15, 30, and 60 min, subsequently reducing the expression levels of c-fos and NFATc1 proteins, effectively inhibiting osteoclast differentiation. Additionally, in the MAPK pathway, phosphorylated STG showed more significant inhibition on phosphorylated JNK, particularly evident at 30, 45, and 60 min, while phosphorylated ERK was also inhibited at 15 and 45 min. Combined with previous research (Xie et al., 2023), our findings deepen the understanding of STG’s mechanisms in osteoclast differentiation, highlighting its significant role. These discoveries not only confirm STG’s function in inhibiting osteoclasts but also support its potential applications in treating osteoporosis.

The Mitogen-Activated Protein Kinases (MAPK) signaling pathway plays a critical role in the differentiation of osteoclasts. This pathway consists of a group of serine/threonine protein kinases that respond to a range of extracellular stimuli. These signals are relayed from the cell membrane to the nucleus, regulating various cellular activities including proliferation, differentiation, survival, and apoptosis (Pearson et al., 2001). Specifically, in the context of osteoclast differentiation, the MAPK pathway finely tunes the maturation process of osteoclast precursors through specific signaling cascades. Activation of the MAPK pathway occurs when osteoclast precursors are stimulated by external factors such as RANKL (Receptor Activator of Nuclear Factor κB Ligand) (Guo et al., 2020). These stimuli engage receptors on the cell membrane to trigger a series of downstream kinases’ phosphorylation, forming a complex signal transduction pathway. Within this pathway, key MAPK subtypes such as p38 MAPK, JNK (c-Jun N-terminal kinase), and ERK (Extracellular signal-regulated Kinase) each possess unique downstream targets and biological effects (Johnson and Lapadat, 2002). The role of p38 MAPK in regulating the activity of osteoclasts and osteoblasts in multiple myeloma cells is a significant area of research. Studies indicate that sustained activation of p38 MAPK in myeloma cells regulates osteoclast differentiation and activity, while concurrently inhibiting osteoblast differentiation, thereby promoting bone destruction. This process involves the expression and secretion of various cytokines, such as DKK-1 and MCP-1, which together promote bone resorption and inhibit bone formation (He et al., 2012). Notably, natural compounds like urolithin A can inhibit the phosphorylation of p38 MAPK, thus suppressing the transmission of the MAPK signaling pathway and reducing the activity of osteoclasts (Li et al., 2022). This finding not only highlights the significant role of the MAPK signaling pathway in osteoclast differentiation but also provides potential therapeutic targets for developing novel treatments for bone diseases.

In summary, our study demonstrated that STG effectively inhibited osteoclastogenesis by modulating NF-κB and MAPK signaling pathways, which in turn suppressed the expression of osteoclast-specific genes such as NFATc1, Acp, and CTSK in Graphical abstract. Moreover, our findings further indicated that oral administration of STG significantly reduced the production of osteoclasts, thereby effectively prevented bone loss induced by OVX. These discoveries provided a novel strategy and insights for the development of STG as an effective drug to treat osteoporosis.

## Data Availability

The original contributions presented in the study are included in the article/supplementary material, further inquiries can be directed to the corresponding authors.
